# Single-cell analysis of human PBMCs in healthy and type 2 diabetes populations: dysregulated immune networks in type 2 diabetes unveiled through single-cell profiling

**DOI:** 10.3389/fendo.2024.1397661

**Published:** 2024-07-12

**Authors:** Doeon Gu, Jinyeong Lim, Kyung Yeon Han, In-Ho Seo, Jae Hwan Jee, Soo Jin Cho, Yoon Ho Choi, Sung Chul Choi, Jang Hyun Koh, Jin-Young Lee, Mira Kang, Dong-Hyuk Jung, Woong-Yang Park

**Affiliations:** ^1^ Department of Health Sciences and Technology, Samsung Advanced Institute for Health Sciences & Technology, Sungkyunkwan University, Seoul, Republic of Korea; ^2^ Samsung Genome Institute, Samsung Medical Center, Seoul, Republic of Korea; ^3^ Department of Biomedical Science, College of Life Science CHA University, Gyeonggi-do, Republic of Korea; ^4^ Department of Health Promotion Center, Samsung Medical Center, Sungkyunkwan University School of Medicine, Seoul, Republic of Korea; ^5^ Department of Digital Health, Samsung Advanced Institute of Health Sciences and Technology (SAIHST), Sungkyunkwan University, Seoul, Republic of Korea; ^6^ Digital Transformation Center, Samsung Medical Center, Sungkyunkwan University School of Medicine, Seoul, Republic of Korea; ^7^ Department of Family Medicine, Yongin Severance Hospital, Gyeonggi-do, Republic of Korea; ^8^ Department of Molecular Cell Biology, Sungkyunkwan University School of Medicine, Seoul, Republic of Korea

**Keywords:** type 2 diabetes, single-cell RNA sequencing, pro-inflammatory characteristics, monocyte, T cell, cell interaction

## Abstract

Abnormalities in glucose metabolism that precede the onset of type 2 diabetes (T2D) activate immune cells, leading to elevated inflammatory factors and chronic inflammation. However, no single-cell RNA sequencing (scRNA-seq) studies have characterized the properties and networks of individual immune cells in T2D. Here, we analyzed peripheral blood mononuclear cells (PBMCs) from non-diabetes and T2D patients by scRNA-seq. We found that CD14 monocytes in T2D patients were in a pro-inflammatory state and intermediate monocytes expressed more MHC class II genes. In T2D patients, cytotoxic CD4 T cells, effector memory CD8 T cells, and γδ T cells have increased cytotoxicity and clonal expansion. B cells were characterized by increased differentiation into intermediate B cells, plasma cells, and isotype class switching with increased expression of soluble antibody genes. These results suggest that monocytes, T cells, and B cells could interact to induce chronic inflammation in T2D patients with pro-inflammatory characteristics.

## Introduction

Diabetes, a prevalent metabolic disorder, affects a substantial portion of the Korean population. According to the Korean Diabetes Fact Sheet 2021, approximately 4.6 million people (13.2%) aged 30 years and above were affected in 2020 (1). Despite a consistent decline in diabetes-related mortality since 2003, it currently ranks as the third leading cause of natural death (2). This chronic condition significantly elevates the risk of various other diseases, stemming from both macrovascular and microvascular damage, thereby exerting detrimental effects on vital organs such as the brain, kidneys, heart, and eyes (3).

In type 2 diabetes (T2D), insulin resistance, resulting from multiple causes, leads to increased insulin secretion to control blood sugar (3). Although early stages of T2D may exhibit normal blood sugar levels due to heightened insulin secretion, abnormal glucose metabolism has already been initiated (4). As T2D progresses, blood sugar levels rise because pancreatic β-cells are unable to secrete sufficient insulin to overcome insulin resistance (4). Moreover, inflammatory factors, including fibrinogen, high-sensitivity C-reactive protein (hsCRP), and IL-6, are elevated in diabetic patients (5). The association of these inflammatory factors with predicting cardiovascular disease among the chronic complications of diabetes is well recognized (6). In addition to their association with macrovascular complications such as cardiovascular disease, recent studies also link inflammatory factors to microvascular complications such as microalbuminuria and diabetic retinopathy (4). In addition to the representative complications of T2D, the chronic inflammatory state of diabetes contributes to elevated risk for various conditions, including cancer, neurodegenerative diseases, depression, and autoimmune disorders (7–10). The constant activation of immune cells could alter their characteristics, creating a vicious cycle that perpetuates the inflammatory state (5).

Single-cell RNA sequencing (scRNA-seq) technology has emerged as the primary method for elucidating the intricate heterogeneity and complexity of RNA transcripts at the single-cell level (11). This advanced approach facilitates the identification and characterization of distinct cell types and provides insights into their unique functions within intricately organized tissues. By employing scRNA-seq, researchers can deepen their understanding of cellular composition and dynamics, shedding light on the intricate workings of biological systems. In type 1 diabetes (T1D), scRNA-seq has been applied to pancreas tissue and peripheral blood mononuclear cells (PBMCs) (12). However, there is an absence of studies analyzing scRNA-seq data using PBMCs from T2D patients. This study aimed to compare PBMCs between T2D patients and non-diabetes, to characterize immune cells and analyze the mechanisms contributing to chronic inflammation in T2D.

## Materials and methods

### Data and code availability

This study was provided with biomedical and research resource, containing genetic and health information from CODA (Clinical & Omics Data Archive), the Agency for Disease Control and Prevention, Republic of Korea (CODA_S2400015-01). Raw files are accessible under the Gene Expression Omnibus (GEO) and accession number is GSE268210. This paper does not report additional code. Any additional information required to reanalyze the data reported in this work paper is available from the lead contact upon reasonable request.

### Study design and participants

PBMCs were obtained from 37 patients diagnosed with T2D, prospectively recruited between August 6th 26th, 2020 and April 3rd, 2021, at the Health Promotion Center of Samsung Medical Center and the Department of Family Medicine of Yongin Severance Hospital. PBMC samples from non-diabetes from the COVID-19 project served as the non-diabetes group.

Type 2 diabetes was defined as any of the following: a fasting plasma glucose level ≥126 mg/dL, a plasma glucose level ≥200 mg/dL at two hours after a 75-g OGTT, an HbA1c ≥6.5%, or current treatment with oral anti-diabetic medications.

This study was approved by the Institutional Review Board (IRB) of Samsung Medical Center (IRB No. 2019–09-121) and by the IRB of Yongin Severance Medical Center (IRB No. 9–2020-0109). All participants provided signed informed consent in accordance with the Helsinki Declaration, allowing for the collection of specimens and detailed analysis of the genetic materials.

### Single-cell isolation

Whole blood samples were collected using BD Vacutainer CPT™ Tubes containing sodium heparin (Cat. No. 362753). PBMCs were isolated from blood samples collected in CPT tubes (maintained at room temperature) within 2–24 hours after blood collection for optimal results. PBMC isolation utilized Ficoll-Paque density gradient centrifugation.

### Genetic multiplexing and sample pooling

Each individual sample was counted and re-suspended to 1.5 × 10^6^ cells/ml in phosphate-buffered saline with 0.04% bovine serum albumin. Equal numbers and volumes of cells from each control were pooled for each experimental batch. Pooled samples were re-counted before being used in the 10x Genomics single-cell experiments.

### 10x scRNA-seq library preparation and processing

For each batch, samples from four controls were pooled. The library preparation utilized a Chromium Next GEM Single Cell 5’Kit v2 (10x Genomics), with a cell recovery target of 4,000 per patient, following the manufacturer’s instructions. Libraries were processed according to the manufacturer’s recommendations (10x Genomics) and sequenced on a HiSeq 2500 system with 100-bp paired-end sequencing. Raw sequencing reads underwent processing using CellRanger (version 5.0.1) (10x Genomics) with default parameter values. The human reference sequence (version GRCh38) and annotation (version GENCODE v32) were employed, utilizing pre-built reference packages provided by 10x Genomics (version 2020-A).

### scRNA-seq data

The scRNA-seq reads underwent processing using CellRanger v5.0.0 to generate the raw expression matrix, comprising unique molecular identifier counts for each gene in every cell. Subsequently, various data preprocessing steps were conducted using the R package Seurat (v4.1.0), encompassing quality control, scaling, transformation, clustering, dimensionality reduction, and visualization. Specifically, cells with fewer than 200 genes and a mitochondrial gene content of less than 5% were excluded from the analysis. For cell type annotation, the LogNormalize function with a scaling factor of 10,000 was applied to normalize the count matrices and identify variable features. To integrate data from different samples and mitigate batch effects, the Harmony method was employed. We used the AddModuleScore function in Seurat(v4.1.0) to score cells for the expression of known gene signatures. Raw files are accessible under the Gene Expression Omnibus (GEO).

### TCR and BCR analysis

The scBCR-seq and scTCR-seq data were assembled using the Cell Ranger VDJ pipeline (v5.0.0, 10x Genomics). Only cells with productive and paired chains, such as IGH and IGL/IGK for BCRs, and TRA and TRB for TCRs, were included in the analysis. In cases where multiple consensus sequences were detected for the same chain type within a cell, all chains were considered for further analysis. The overall clonality of the sample’s repertoire was assessed using the Gini coefficient, which measures the degree of inequality among clonotypes.

### Pseudobulk analysis

We performed pseudobulk analysis by treating the single-cell RNA profiles as pseudobulk expression matrices to evaluate the trend of pro-inflammation genes among each patient. We ran DEseq2 on the aggregated pseudobulk gene expression data. We next formed a correlation matrix using Spearman’s correlation across the expression matrix containing only pro-inflammation genes (CXCL8, CCL2, CCL3, CCL5, IL1B, CXCL9, and CXCL10), and clustered the samples using hierarchal clustering.

### Enrichment analysis

For the identification of marker genes specific to each status (T2D and non-diabetes samples), we utilized the Seurat FindMarkers function. Signature genes were required to be expressed in >25% of cells within either of the two cell groups. The selection of differentially expressed genes (DEGs) was based on a statistical threshold (avg_log2FC > 0.5 and *p*-value < 0.05). To gain insights into cellular pathways associated with the selected DEGs, we performed gene set enrichment analysis (GSEA) using the clusterProfiler (V4.6.2) package, with the GO Biological Process dataset as the reference dataset.

### Cell-to-Cell communication

We performed Cell-to-Cell communication analysis using ChellChat R package (V 1.4.0) which can then be used for comparative analysis across multiple datasets. CellChat enables for comparing the number and strength of interactions among various cell populations, computing signals for each cell population, and screening essential ligand/receptor pairs across different cells.

### Statistical analysis

R statistical software (version 4.0.2) was used for statistical analysis. The data were reported as mean ± SEM. An unpaired two-tailed t-test was performed for experiments to test significance between the two groups.

## Results

### scRNA-seq identified an altered immune cell atlas in T2D

**Figure 1:**
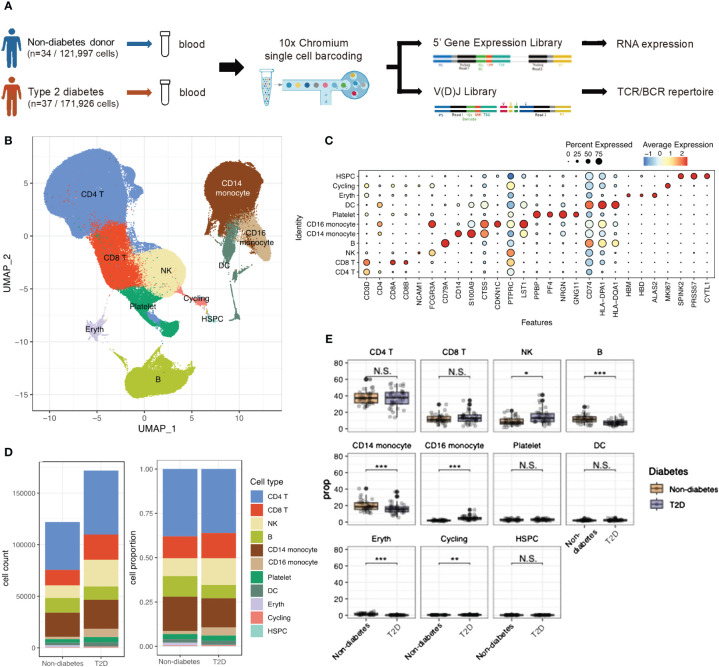
scRNA-seq identified Type 2 diabetes immune cell atlas. **(A)** Schematic representation of experimental design and techniques used in this study. 34 non-diabetes PBMC samples and 37 type 2 diabetes patients were collected for analysis. **(B)** UMAP plot of 293,923 PBMC cells shows the major cells types. Each dot represents a single cells, and colors represent different cell types. **(C)** Dot plot of mean expression of canonical markers across the cell types. **(D)** The proportion of global cell clusters depending on normal or diabetes using bar plot. **(E)** The proportion of global cell clusters depending on normal or diabetes using box plot. N.S. not significant, *p < 0.05, **p < 0.01, ***p < 0.001.

The scRNA-seq datasets were obtained from PBMCs of 34 non-diabetes and 37 T2D patients, aiming to investigate the cellular composition of PBMCs in T2D (**Figure 1A**). For comparability, we matched non-diabetes and T2D patients based on sex, age, and BMI, with only a slight BMI difference among females due to the strong association between BMI and diabetes. **Table 1** shows the baseline characteristics of the 34 non-diabetes and 37 T2D patients. Age, sex, and BMI did not show differences between the non-diabetes and T2D patients. The following variables were analyzed in the T2D patients and were not obtained during the sample recruitment process in the non-diabetes: HbA1c, glucose, total cholesterol, triglyceride, HDL, and LDL.

**Table 1:** Baseline characteristics of the study population.

	Healthy controls	Type 2 diabetes	P-value
N	34	37	
Age	51 (12)	56 (7)	0.14
Sex			0.4
Female	16 (47 %)	14 (38 %)	
Male	18 (53 %)	23 (62 %)	
BMI	25.0 (3.2)	26.4 (3.3)	0.14
Glucose	NA	133 (27)	
HbA1c	NA	6.5 (2.8)	
Total cholesterol	NA	150 (35)	
Triglyceride	NA	127 (68)	
HDL	NA	52 (13)	
LDL	NA	97 (40)	
AST	NA	34 (17)	
ALT	NA	31 (17)	

Data are expressed as the mean (SD), or percentage.

P values were calculated using Wilcoxon rank sum test, and Pearson’s Chi-squared test.

After filtering out low-quality cells, a total of 293,923 PBMCs were analyzed. Violin plots before quality control (QC) and after filtering was shown in **Supplementary Figures 1A, B**. Uniform Manifold Approximation of Projection (UMAP) was employed to delineate the T2D PBMC atlas (**Figure 1B**). Integrating scRNA-seq data using the Harmony method revealed no obvious specimen-derived bias. Clustering analysis identified 11 major cell types annotated by marker genes, including CD4 T cells (CD3D, IL7R), CD8 T cells (CD3D, CD8B), NK cells (NCAM1), B cells (CD79A), CD14 monocytes (CD14, S100A9), CD16 monocytes (CDKN1C), platelets (PPBP), dendritic cells (DCs; CD74), erythrocytes (HBM), cycling cells (MKI67), and hematopoietic stem and progenitor cells (HSPCs; SPINK2) (**Figure 1C**). **Supplementary Figure 1C** showed the PBMC atlas by each patient, non-diabetes or T2D patients, and sex, respectively. The cell number and cell population for each subject could be found in **Supplementary Figure 1D**. The global cell type feature plot is shown in **Supplementary Figure 1E**.

Differences in cell type composition were observed between the non-diabetes and T2D patients (**Figures 1D, E**). T2D patients showed higher proportions of NK cells and CD16 monocytes, while B cells and CD14 monocytes were significantly more abundant in non-diabetes.

### T2D monocytes exhibited heightened expression of cytokine and antigen-presenting genes

**Figure 2:**
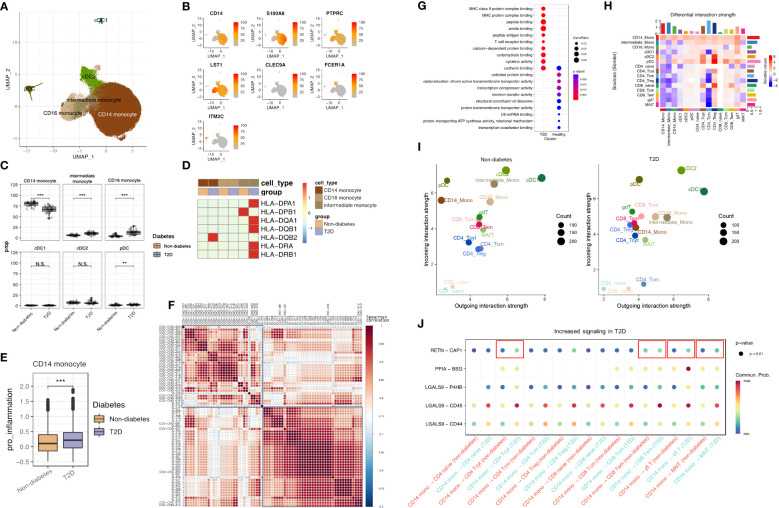
T2D monocytes have increased expression of inflammatory molecules and antigen-presenting genes. **(A)** The UMAP visualization shows the distribution of 56,250 myeloid cells, revealing the presence of six distinct monocyte cell clusters, as well as clusters representing dendritic cells. **(B)** The expression of myeloid cell marker genes is depicted in the UMAP plot, highlighting their expression patterns across the identified myeloid clusters. **(C)** The proportion of myeloid clusters depending on normal or diabetes **(D)** The expression pattern of cytokine gene and MHC class II genes in monocytes. **(E)** Distributions of CD14 Mono pro-inflammation module score with respect to normal and diabetes. **(F)** Different patterns heatmap between non-diabetes and T2D patients by pro-inflammatory genes (CXCL8, CCL2, CCL3, CCL5, IL1B, CXCL9, and CXCL10) using pseudobulk differential expression analysis **(G)** The top 10 enriched biological processes by GO analysis of upregulated DEGs of CD14 Mono of diabetes and normal group. Dot color indicates the statistical significance of enrichment and dot is in proportion of gene ratio of the enriched gene number. **(H)** Heatmap showing the differential number of interactions between non-diabetes and T2D patient. In the color bar, red (or blue) represents increased (or decreased) signaling in the T2D patient compared to non-diabetes. **(I)** Scatter plots comparing the outgoing and incoming interaction strength in the 2D space among each cell population. **(J)** Bubble chart of cell to cell signaling in non-diabetes and T2D patient. N.S. not significant, **p < 0.01, ***p < 0.001.

To explore transcriptome dynamics in 56,260 cells identified as monocytes and DCs, we subclustered monocytes into three subsets and DC into three subsets using canonical gene markers: CD14 monocytes (expressing CD14 and S100A8), CD16 monocytes (expressing PTPRC and LST1), intermediate monocytes displaying characteristics of both CD14 monocytes and CD16 monocytes, conventional DCs type 1 (cDC1s; expressing CLEC9A), cDC2s (expressing FCER1A), and plasmacytoid DCs (pDCs; expressing ITM2C) (**Figures 2A, B**). Composition differences emerged between non-diabetes and T2D patients. The proportion of CD14 monocytes significantly decreased in T2D, while intermediate monocytes and CD16 monocytes increased in T2D (**Figure 2C**).

Validation through the expression of MHC class II genes (**Figure 2D**) showed that these genes were most highly expressed in intermediate monocytes in T2D, consistent with previous research indicating the significant expression of antigen presentation-related molecules in this subset (13).

A pro-inflammation score, calculated for CD14 monocytes based on the expression of pro-inflammatory genes (CXCL8, CCL2, CCL3, CCL5, IL1B, CXCL9, and CXCL10) with the AddModuleScore function, revealed differing subsets in pro-inflammation scores between T2D and non-diabetes, with T2D displaying a higher pro-inflammation score (**Figure 2E**). Further analysis of CD14 monocytes by pseudobulk differential expression analysis, the expression of pro-inflammatory genes differentiated between non-diabetes and T2D patients and were highly expressed in T2D patients (**Figure 2F**). Gene Ontology (GO) enrichment analysis, specifically on CD14 monocytes (**Figure 2G**), highlighted pathways associated with T2D, including MHC class II protein complex binding, MHC protein complex binding, T-cell receptor binding, and cytokine binding. Sending signaling of CD14 monocytes was increased in T2D (**Figure 2H**) and the outgoing interaction strength of CD14 monocytes was higher in T2D patients than in non-diabetes (**Figure 2I**). Furthermore, RETN and CAP1 interaction, which are associated with inflammation in T2D (14), was increased between CD14 and CD4 cytotoxic T cell (CD4 Tcyt), CD14 and CD8 effector memory (CD8 Tem), CD14 and γδ T cell (γδT), and CD14 and MAIT (**Figure 2J**).

These findings collectively indicate heightened activity in CD14 and intermediate monocytes in T2D conditions, reflecting a more activated immune system in individuals with T2D compared to those without the condition. In particular, CD14 monocytes have inflammative signature and play a role in inflammation with T cells and interacted with other cells through sending signals. Furthermore, the increased expression of MHC class II genes in intermediate monocytes underscores their crucial role in antigen presentation to CD4 T cells and their potential to recruit T cells.

### T2D elicited enhanced cytotoxicity and clonal expansions of cytotoxic T cells

**Figure 3:**
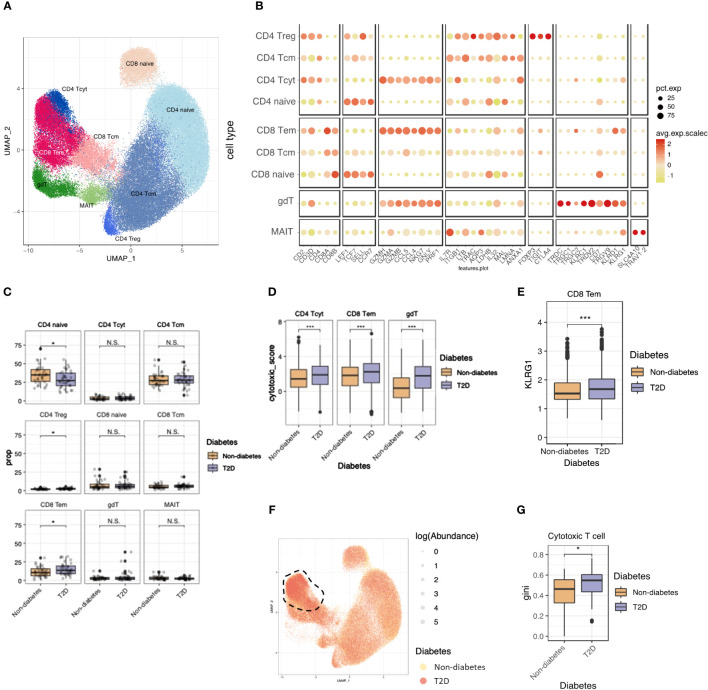
T2D enhanced cytotoxicity and clonal expansions of cytotoxic T cells. **(A)** The UMAP visualization shows the distribution of 21,871 T cells, revealing the presence of five distinct T cell clusters, as well as clusters representing NK cells. **(B)** Dot plot of mean expression of canonical markers across the cell types. **(C)** The proportion of T cell clusters depending on normal or diabetes. **(D)** Distributions of CD4_Tcyt, CD8_Tem cytotoxic and gdT module score with respect to normal and diabetes. **(E)** Distributions of CD8_Tem KLRG1 expression score with respect to normal and diabetes. **(F)** The UMAP plot specifically focuses on the visualization of marked TCR abundance. The circle size represents the log(Abundance) and color represent normal and diabetes. **(G)** Distributions of Gini index score of cytotoxic T cells (CD4_Tcyt and CD8_Tem) depending on normal and diabetes. N.S. not significant, *p < 0.05, ***p < 0.001.

Continuing our investigation into T cells influenced by monocytes, we observed a shift in the proportions of T-cell populations, notably marked by an increase in CD8 T cells. A re-clustering of T cells with canonical markers revealed nine subtypes, including CD4 naïve, CD4 central memory (CD4 Tcm), CD4 regulatory T cell (CD4 Treg), CD4 cytotoxic T cell (CD4 Tcyt), CD8 naïve, CD8 central memory (CD8 Tcm), CD8 effector memory (CD8 Tem), γδ T cell (γδT), and MAIT cells (**Figures 3A, B**). **Supplementary Figure 1F** shows the expression of T cells marker genes in UMAP plot. T-cell composition varied between non-diabetes and T2D patients, with a significant decrease in CD4 naïve cells in T2D patients compared to non-diabetes. (**Figure 3C**).

To assess the functional aspects of cytotoxic T cells, we calculated the cytotoxicity score for CD4 Tcyt and CD8 Tem using cytotoxic genes (PRF1, GZMH, and GZMK) and observed higher cytotoxicity scores in T2D patients compared to non-diabetes (**Figure 3D**). Glucose metabolism plays a crucial role in regulating the effector killing function of T cells. This implies that the hyperglycemic conditions observed in T2D may enhance the cytotoxicity of CD4 Tcyt and CD8 Tem cells (15, 16).

We examined T-cell senescence by analyzing KLRG1 expression, a marker associated with senescence in T cells (17). In CD8 Tem cells, KLRG1 expression was elevated in individuals with T2D compared to non-diabetes (**Figure 3E**). This suggests that CD8 Tem cells in T2D are not only more activated than their normal counterparts but also exhibit a faster senescent state compared to normal conditions.

To explore clonal relationships among individual T cells, T cell receptor (TCR) analysis was performed across the nine subsets. The percentage of matched TCRs with scRNA-seq was 81.6%. In T2D patients, there were significant clonal expansions observed in cytotoxic T cells (CD4 Tcyt and CD8 Tem) (**Figure 3F**), with the clonality size, measured using the Gini index, being notably higher in T2D cytotoxic T cells than in those from the non-diabetes (**Figure 3G**). These heightened-cytotoxicity and clonality features in cytotoxic T cells contribute to the pro-inflammatory immune environment associated with T2D.

### T2D enhanced B-cell differentiation and isotype class switching

**Figure 4:**
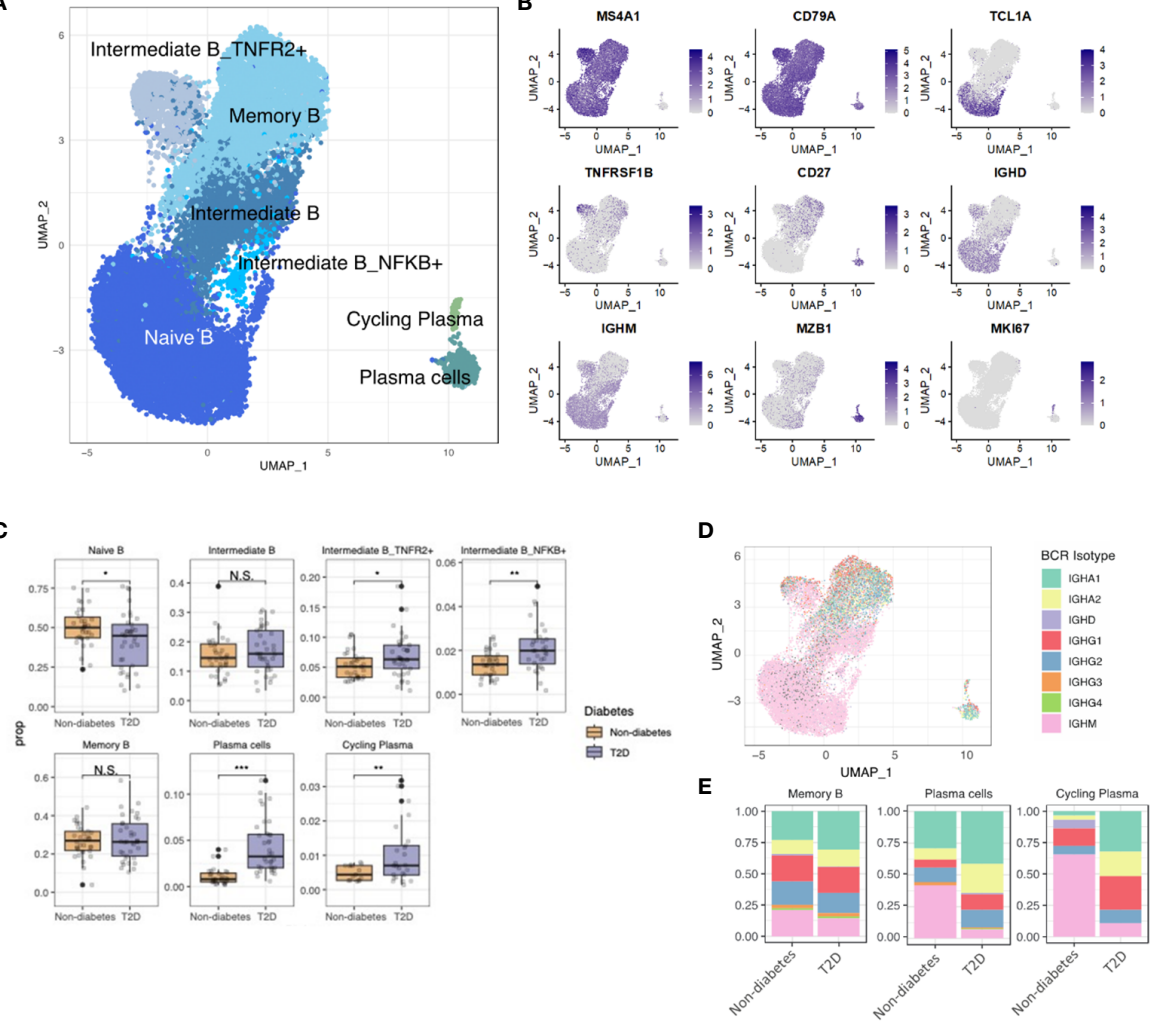
Increased Plasma Cell Differentiation and BCR diversity in Diabetes. **(A)** The UMAP visualization shows the distribution of 21,871 B cells, revealing the presence of five distinct B cell clusters, as well as clusters representing plasma cells and cycling plasma cells. **(B)** The expression of B cell marker genes is depicted in the UMAP plot, highlighting their expression patterns across the identified B cell clusters. **(C)** The proportion of B cell clusters depending on normal or diabetes. **(D)** The UMAP plot specifically focuses on the visualization of marked B cell receptor (BCR) isotypes. The color-coding indicates the presence or absence of matched BCR data, with grey color representing cases where BCR data was not available. **(E)** The distribution of BCR isotypes within each B cell and plasma cell cluster based on the normal or diabetes. N.S. not significant, *p < 0.05, **p < 0.01, ***p < 0.001.

To elucidate B-cell characteristics in T2D patients, we investigated five distinct B-cell subsets and two clusters of plasma cells. These subsets included naïve B cells (expressing MS4A1, CD79A, TCL1A, IGHM, and IGHD), memory B cells (expressing MS4A1, CD79A, and CD27), plasma cells (expressing MZB1), and intermediate B cells (exhibiting characteristics of both naïve and memory B cells and expressing TNFR1B and NKFB) (**Figures 4A, B**).Our observations revealed an elevated proportion of intermediate B cells (identified as intermediate B_TNFR2+ and intermediate B_NFKB+) and plasma cells, while the naïve B-cell proportion was lower in T2D patients than the non-diabetes (**Figure 4C**). Additionally, we analyzed B-cell receptor (BCR) data, comparing T2D patients and non-diabetes. The findings indicated that individuals with T2D exhibited a higher degree of differentiation into plasma cells and increased diversity of BCR isotypes (**Figures 4D, E**). Overall, our results suggest that T2D is associated with changes in B-cell subsets, including an expansion of intermediate B cells, increased differentiation into plasma cells, and heightened isotype class switching in memory B cells. These alterations may contribute to the pro-inflammatory immune environment associated with T2D, similar to the role of T cells.

## Discussion

T2D induces systemic chronic inflammation, heightening the risk of cancer, cardiovascular diseases, and autoimmune conditions. However, scRNA-level analysis of immune cells orchestrating chronic inflammatory states has predominantly focused on T1D. This study illustrated that inflammation-related alterations within individual immune cell populations are more pronounced in T2D patients than in non-diabetes. Specifically, our findings highlight heightened MHC class II protein expression and inflammatory interaction in T2D monocytes. Additionally, CD4 and CD8 T cells exhibited increased cytokine scores and clonality, while differentiated B cells, including TNFR2+ intermediate B cells, NFKB+ intermediate B cells, and plasma cells, showed increased proportions and engaged in antibody isotype switching to IgG and IgA. Our study underscores the interconnectedness and interaction among immune cell populations in T2D, suggesting a potential induction of chronic inflammation and susceptibility to autoimmune diseases. The study of Dora et. al (18). that CD8 and γδ T cells in T2D make many molecules that increase inflammaging support our study. In addition to those commonalities, we showed that other cells, such as, CD14 monocytes, CD4 T cells, and B cells, also have inflammatory properties.

Monocytes constitute a crucial component of the innate immune system, playing a role in regulating cellular homeostasis and inflammatory processes (19, 20). T2D patients exhibit a reduction in CD14 monocytes (classical monocytes), which differentiate into M2 macrophages with anti-inflammatory characteristics (21). However, in the context of T2D, classical monocytes are vulnerable and functionally impaired under endoplasmic reticulum stress (22).

In this study, T2D patients demonstrated a decreased proportion of CD14 monocytes and an increased proportion of intermediate and CD16 monocytes compared to non-diabetes.

Interestingly, our current study revealed that CD14 monocytes in T2D exhibited a significantly higher pro-inflammatory score and enriched pathways associated with MHC class II protein complex binding and RETN-CAP1 interaction compared to the non-diabetes. This suggests that while CD14 monocytes in T2D could display inflammatory characteristics. Furthermore, intermediate monocytes in T2D patients demonstrated an upregulation of MHC class II molecule genes compared to the non-diabetes. These findings in T2D patients may contribute to the chronic inflammatory status, with the upregulation of MHC class II molecules potentially influencing T-cell activity.

In the current study, the cytotoxic scores of CD4 Tcyt cells, CD8 Tem cells, and γδ T cells were higher in T2D patients than in the non-diabetes. Our findings in monocytes and T cells could provide a mechanism for the persistence of the chronic inflammatory state in T2D patients through MHC class II and an antigen-presenting cell (23).

We next delved into the expression change of cytotoxic genes in CD4 Tcyt, CD8 Tem, and γδ T cells (24, 25). Previous studies, particularly in T1D, have implicated granzyme and perforin secreted by CD8 T cells in the destruction of β cells (26). Furthermore, increased serum levels of granzyme B have been independently associated with T2D diagnosis (27), underscoring the potential significance of CD4 Tcyt cells in the pathophysiology of diabetes. In a T1D mouse model, macrophages and CD4 T cells developed into a pro-inflammatory subtype (28). Comprehensive analyses showed that the expression and chromatin accessibility of cytotoxic genes in CD4 T cell were heightened in children with T1D (29).

In our study, we observed an increase in the cytotoxicity of CD4 T cells, CD8, and γδ T cells. These findings suggest crucial roles for T cells in the pathophysiology of T2D. Additionally, we identified heightened expression of KLRG1 on CD8 Tem cells in TD2, reflecting the senescent status of T cells (30). Furthermore, our study demonstrated clonal expansions of cytotoxic T cells in T2D patients than in the non-diabetes. Exhibiting clonal expansion and cytotoxicity of senescent T cells suggests that persistently stimulated T cells potentially contribute to chronic inflammation in T2D.

In the context of B-cell development into plasma cells, the involvement of CD4 T cells is crucial (23). Our study revealed a reduction in naïve B cells and an increase in TNFR2+ intermediate B cells, NF-κB+ intermediate B cells, and plasma cells in T2D patients. NF-κB, a pivotal signaling molecule, plays a significant role in B-cell activation and development by inhibiting B-cell apoptosis and influencing peripheral B-cell survival (31, 32). Plasma cells, derived from B cells with support from CD4 T cells, can produce large amounts of antibodies (33). In T2D patients, we observed an elevation in plasma cells, coupled with changes in antibody isotypes, resulting in increased secretion of IgA and IgG antibodies. While IgA plays a protective role on mucosal surfaces, its increased expression during the inflammatory phase of T2D may induce various immune cells to express the Fc receptor in multiple tissues, promoting the production of pro-inflammatory cytokines (34). Elevated IgG levels are associated with various autoimmune diseases and the level of procytokines, and our findings align with various (35, 36).

Our study showed that the sequential activation of monocytes and CD4 T cells influences B-cell development perpetuation of the pro-inflammatory status observed in T2D. However, the increased population of TNFR2+ intermediate B cells may have a counteractive effect through IL-10, anti-inflammatory cytokine (37). The observed increase in TNFR2+ intermediate B cells could be a complementary consequence of an excessively inflammatory status. Further investigation is needed to determine whether TNFR2+ intermediate B cells indeed possess anti-inflammatory effects in the context of T2D.

When we analyzed correlation between our findings and the clinical characteristics of T2D patients, the inflammation score of CD14 monocytes was correlated with BMI, but the correlation coefficient was small. (R=0.36, p=0.037) (**Supplementary Figure 2**). These results suggest that due to the fact that T2D patients are taking medications to control their disease, so they do not show significant differences in clinical characteristics, but rather changes at the cellular level. Further research is needed to confirm the results of this study using flow cytometry to determine the actual proportion of and the function of the immune cells.

The information presented in **Table 1**-- was limited because the prevalence of T2D was determined using a questionnaire during the recruitment process for non-diabetes participants. However, in Korea, the prevalence of T2D could be determined through regular national and employee medical checkups, so non-diabetes participants in this study could be classified as not having T2D. Second, the medication information of T2D patients was not presented. Further studies are needed to provide more accurate information on non-diabetes participants and more detailed comparisons including medication information for T2D. Despite these limitations, our findings revealed intricate interactions among monocytes, T cells, and B cells, collectively contributing to the establishment of a chronic inflammatory state in T2D patients. These results emphasize the potential heightened risk of cancer, cardiovascular disease, and autoimmune diseases among T2D patients due to the sustained presence of chronic inflammation.

## Data availability statement

The datasets presented in this study can be found in online repositories. The names of the repository/repositories and accession number(s) can be found in the article/Materials and methods.

## Ethics statement

The studies involving humans were approved by Institutional Review Board (IRB) of Samsung Medical Center (IRB No. 2019-09-121) and by the IRB of Yongin Severance Medical Center (IRB No. 9-2020-0109). The studies were conducted in accordance with the local legislation and institutional requirements. The participants provided their written informed consent to participate in this study.

## Author contributions

DG: Formal analysis, Writing – original draft, Writing – review & editing. JL: Validation, Writing – original draft, Writing – review & editing. KH: Formal analysis, Writing – original draft, Writing – review & editing. I-HS: Formal analysis, Writing – original draft, Writing – review & editing. JJ: Supervision, Writing – review & editing. SoC: Supervision, Writing – review & editing. YC: Supervision, Writing – review & editing. SuC: Supervision, Writing – review & editing. JK: Supervision, Writing – review & editing. J-YL: Supervision, Writing – review & editing. MK: Supervision, Writing – review & editing. D-HJ: Writing – original draft, Writing – review & editing. W-YP: Writing – original draft, Writing – review & editing.
