# Supplementary figures and images for: Single-cell analysis of human PBMCs in healthy and type 2 diabetes populations: dysregulated immune networks in type 2 diabetes unveiled through single-cell profiling
Source: Front Endocrinol (Lausanne). 2024 Jul 12;15:1397661. doi: 10.3389/fendo.2024.1397661 (PMC11272961; doi:10.3389/fendo.2024.1397661)

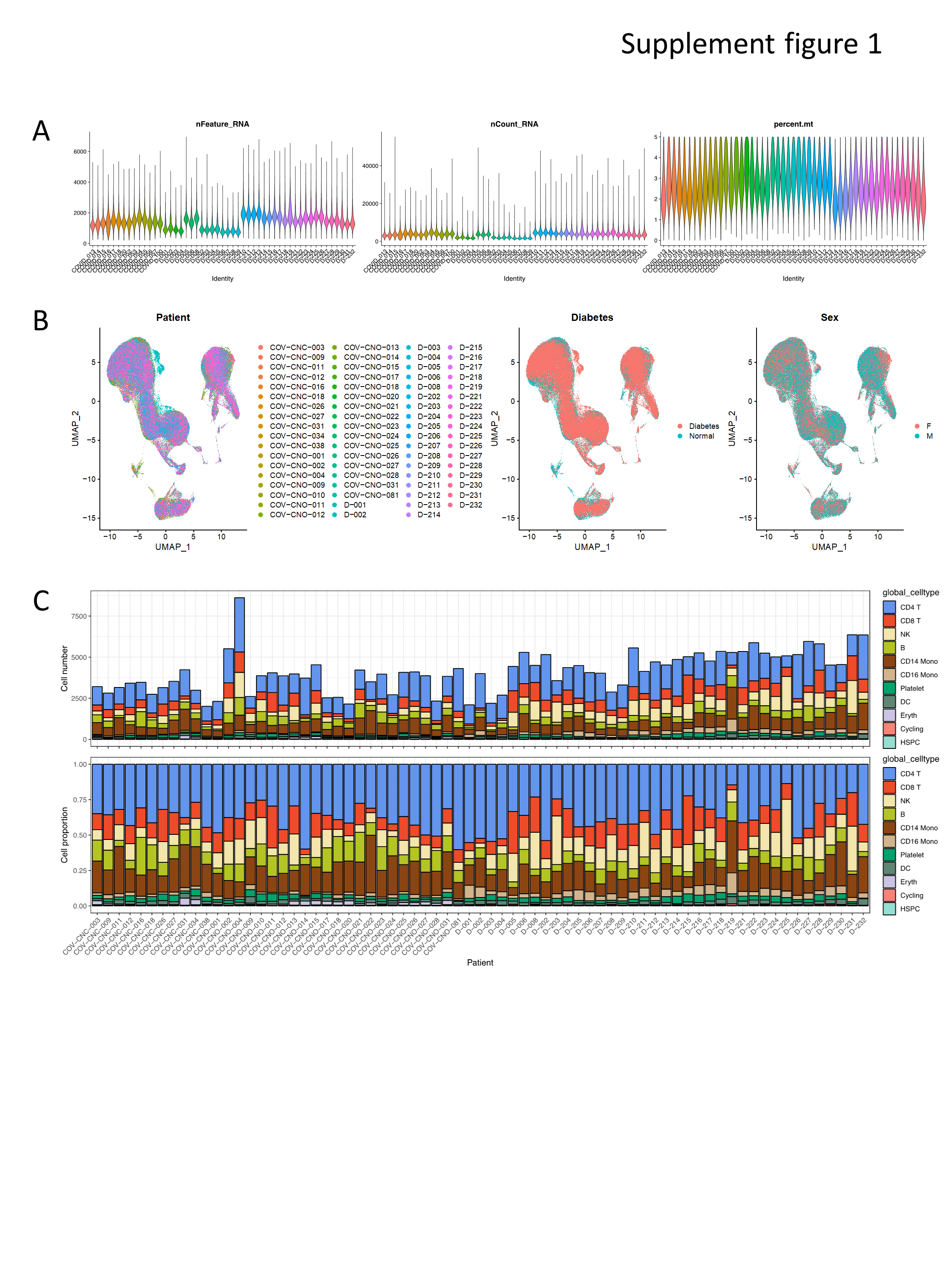

Supplement: Supplementary file 1 [file Image_1.tif]

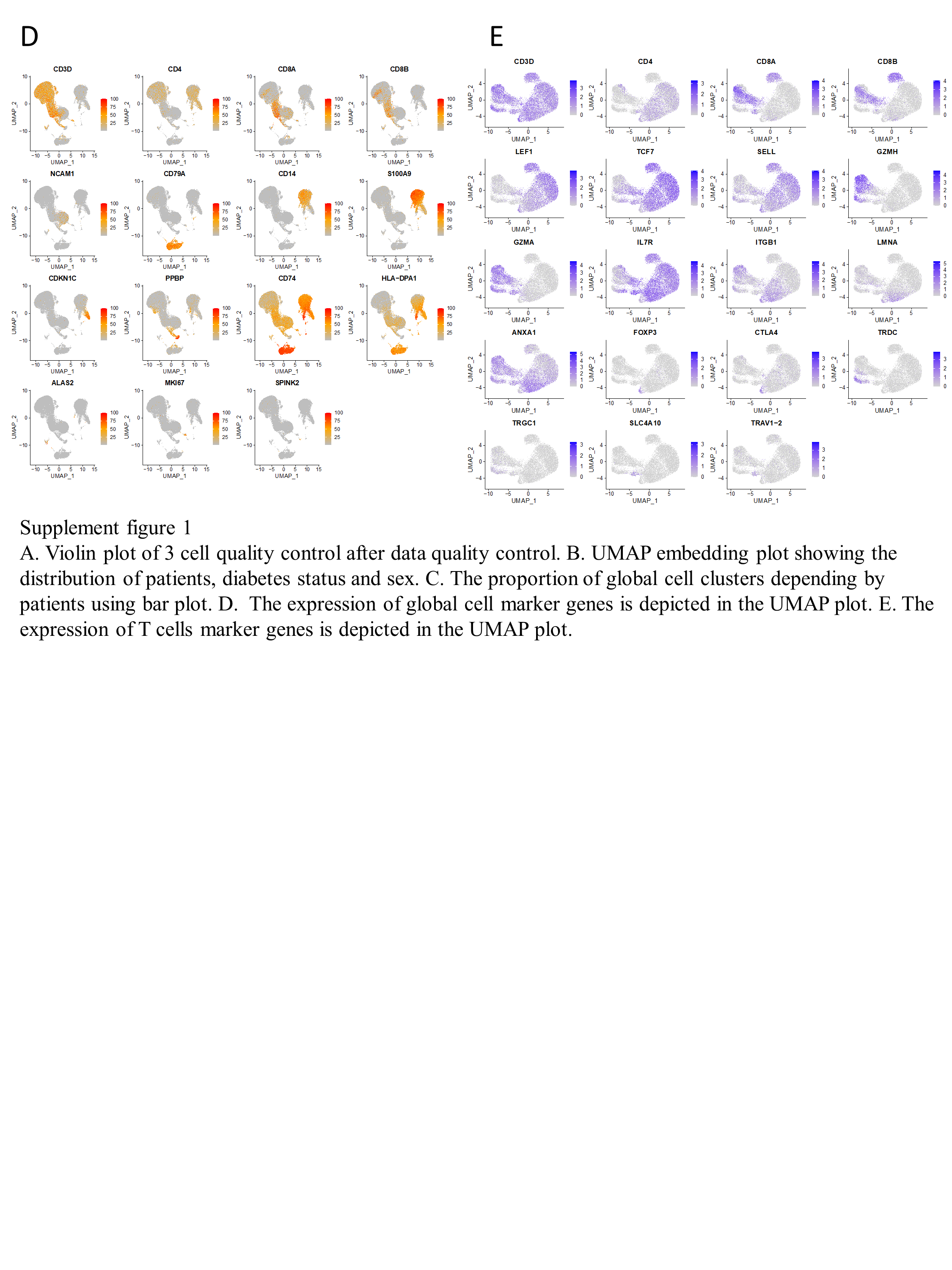

Supplement: Supplementary file 2 [file Image_2.tif]

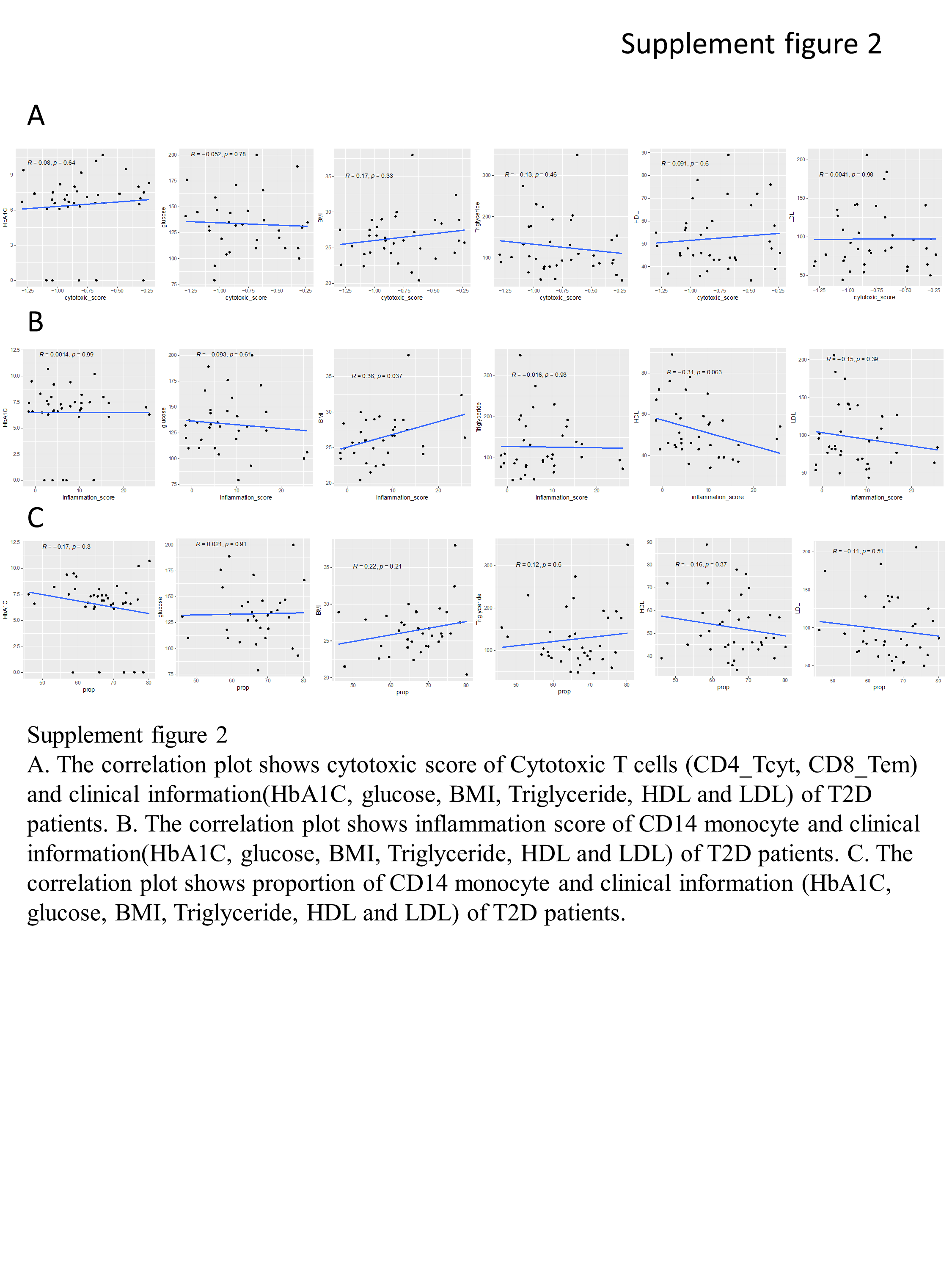

Supplement: Supplementary file 3 [file Image_3.tif]
